# The value of distinct depressive symptoms (PHQ‐9) to differentiate depression severity in cancer survivors: An item response approach

**DOI:** 10.1002/pon.5192

**Published:** 2019-08-30

**Authors:** Loek J. van der Donk, Esmée A. Bickel, Wim P. Krijnen, K. Annika Tovote, Robbert Sanderman, Maya J. Schroevers, Joke Fleer

**Affiliations:** ^1^ Department of Health Psychology, University Medical Center Groningen University of Groningen Groningen the Netherlands; ^2^ Research Healthy Ageing Allied Health Care and Nursing Hanze University of Applied Sciences Groningen Groningen the Netherlands; ^3^ Department of Psychology, Health and Technology University of Twente Enschede the Netherlands

**Keywords:** cancer, depression, discriminative capacities, item response theory, oncology, PHQ‐9, screening, somatic overlap

**Key points**

- The Patient Health Questionnaire‐9 (PHQ‐9) is frequently used for detecting depressive symptoms in the general population as well as in an oncology setting.
- Screening for depressive symptoms among cancer survivors is challenged by the overlap of somatic depression symptoms with symptoms of cancer and its treatment.
- No study has examined the discriminative capacities of distinct PHQ‐9 depressive symptoms.
- We found that all PHQ‐9 items (both cognitive‐affective and somatic) have excellent discriminative capacities for measuring depressive symptoms among cancer survivors.
- Somatic symptoms, in particular fatigue and sleep problems, seem to best discriminate for mild depression, whereas cognitive‐affective symptoms discriminate best for moderate to severe depression.

## 1 INTRODUCTION

Depressive symptoms are common among cancer patients and may persist after finishing curative treatment. Typical depressive symptoms include cognitive‐affective symptoms, such as having a depressed mood and somatic symptoms such as fatigue^1^. It is well known that somatic symptoms of depression may overlap with symptoms of the illness and its treatment, hereby increasing the risk of false‐positive diagnoses.^1^, ^2^ Whether or not to include somatic symptoms in the assessment of depressive symptoms in cancer patients has therefore been an on‐going debate over the past decades,^1^, ^2^ with recent studies suggesting to include somatic symptoms also in persons with a somatic condition.^1^, ^2^, ^3^, ^4^, ^5^

A limitation of previous studies addressing the inclusion or exclusion of somatic symptoms in the assessment of depressive symptoms is that they focused on *domains* of symptoms, that is, to include or exclude *all* somatic symptoms and *all* cognitive‐affective symptoms. By doing so, they overlooked the role of *distinct* depression symptoms as was recommended more recently,^2^, ^6^ given the heterogeneity with in the somatic or cognitive/affective domain.^6^ Another limitation is the focus on “depressed” versus “not depressed”,^1^, ^5^ with a call for more research addressing the continuum of depression severity.^4^

Following these recommendations to focus on distinct depressive symptoms and the continuum of depression severity, we examined how distinct cognitive‐affective and somatic symptoms of depression are related to the continuum of depression severity and how these symptoms discriminate different levels of depression severity, using an item response approach.

## 2 METHOD

### 2.1 Procedure

For the current paper, we used data collected during routinely depression screening in clinical practice, which was part of a larger intervention trial on the effectiveness of depression treatment among cancer survivors.^7^ Patients were routinely screened on depressive symptoms using the Patient Health Questionnaire‐9 (PHQ‐9). Those eligible and willing to participate were asked to fill in an informed consent form prior to trial participation. Inclusion criteria were any cancer diagnosis except for breast cancer, age 18‐75 at time of diagnosis, no active cancer, and having completed curative cancer treatment at least 1 year ago up to 5 years ago. The study was approved by the Medical Ethical Committee of the University Medical Center Groningen.

### 2.2 Measures

The PHQ‐9 is a valid self‐report instrument based on the DSM‐IV diagnosis of a depressive disorder,^8^ consisting of nine items that can be scored from 0 to 3, resulting in a total score between 0 and 27. Depression severity categories are no (0‐4), mild,^5^, ^6^, ^7^, ^8^, ^9^ moderate (10‐14), moderately‐severe (15–19), and severe depression (20–27).^8^ Reliability was good (α = 0.87 and ω = 0.89) in our study.

### 2.3 Statistical analysis

For demographic and clinical variables, means and percentages were calculated using SPSS 23.0. The R programming language was used for conducting item response theory analyses using package KernSmoothIRT. To examine how distinct cognitive‐affective and somatic symptoms of depression are related to the continuum of depression severity, we used polyserial correlation coefficients to estimate the association between ordinal items and the continuous overall depression trait. To examine how distinct symptoms discriminate different levels of depression severity, we plotted expected item scores against the expected values on the overall depression trait, in order to localize the most discriminative areas.

## 3 RESULTS

In total, 2099 cancer survivors (74.6% of the approached 2814) returned the questionnaire. Mean age was 63.39 years (±10.27) of which 62% was male. Average years since diagnosis was 3.32 (±1.31), and years since treatment was 2.99 (±1.25). Most common cancer types were gastrointestinal (39.6%) and urological tumors (19.2%) with surgery only (21.7%) or radiotherapy only (20.4%) as most received treatments. The response distributions of the nine PHQ‐9 items differed considerably among items (see Table 1), with most commonly reported symptoms being “sleep problems” (39.2%) and “fatigue” (45.9%), whereas “suicidality” was only endorsed by 5.5% of the cancer survivors.

**Table 1:** Descriptive statistics and polyserial correlation coefficients for the nine Patient Health Questionnaire‐9 (PHQ‐9) items

		Answer Categories, %				Polyserial Correlations	
PHQ‐9 Item	Mean ± SD	Not at all	Several days	More than half the days	Nearly every day	Coefficients	CI
Item 1 (Anhedonia)	0.33 ± 0.69	76.3	16.9	4.0	2.8	0.77	0.74‐0.80
Item 2 (Depressed mood)	0.23 ± 0.56	82.1	14.1	2.4	1.4	0.79	0.76‐0.81
Item 3 (Sleep problems)	0.63 ± 0.94	60.8	23.7	6.9	8.6	0.73	0.71‐0.76
Item 4 (Fatigue)	0.72 ± 0.95	54.1	28.9	8.0	9.1	0.80	0.78‐0.82
Item 5 (Appetite change)	0.26 ± 0.66	83.8	9.5	4.0	2.8	0.70	0.66‐0.74
Item 6 (Low self‐esteem)	0.18 ± 0.53	87.4	8.9	2.2	1.5	0.74	0.69‐0.77
Item 7 (Concentration difficulty)	0.30 ± 0.67	79.0	14.6	3.6	2.8	0.71	0.67‐0.75
Item 8 (Psychomotor)	0.16 ± 0.52	89.6	6.3	2.7	1.4	0.63	0.58‐0.67
Item 9 (Suicidality)	0.07 ± 0.33	94.5	4.2	0.9	0.4	0.59	0.54‐0.64

Abbreviations: CIs: confidence intervals; SD: standard deviation.

### 3.1 Associations of distinct depressive symptoms (PHQ‐9) with depression severity

Polyserial correlations were computed to estimate the association between the nine items and the continuous overall latent depression trait (Table 1). Largest correlations were found for “fatigue” (0.80), “depressed mood” (0.79), and “anhedonia” (0.77), indicating that these symptoms are most strongly associated with the latent depression trait. The weakest correlation was found for “suicidality” (0.59).

### 3.2 Discriminative capacities of distinct depressive symptoms (PHQ‐9)

**Figure 1:**
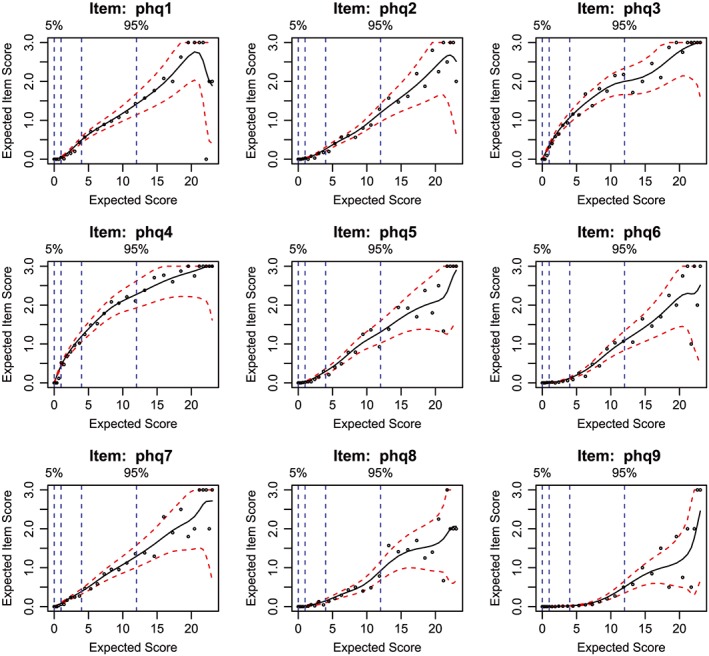
Expected item scores as a function of depression severity for all items of the Patient Health Questionnaire‐9 (PHQ‐9)

The expected item scores revealed that all items showed monotonicity (Figure 1), with increased item scores being associated with increased depression severity. In general, the complete set of nine items covered the continuum of depression severity reasonably well. Item 3 “sleep problems” and item 4 “fatigue” display concave slopes, indicating particularly high discrimination for mild depressive symptoms, whereas item 8 “psychomotor problems” and item 9 “suicidality” have more convex slopes, indicating high discrimination for moderately severe depressed individuals. Item 1 “anhedonia,” item 2 “depressed mood,” item 5 “appetite changes,” item 6 “low self‐esteem,” and item 7 “concentration difficulty” show constant linear slopes, indicating that these items discriminate evenly well for mild, moderate, and severe depression levels.

## 4 DISCUSSION

Our results showed that the two core symptoms (ie, depressed mood and anhedonia) and fatigue were most strongly associated with the latent depression trait. Overall, all distinct PHQ‐9 symptoms showed excellent discriminative capacities among cancer survivors, with somatic symptoms (ie, sleep problems and fatigue) discriminating best for mild depression and psychomotor problems and suicidal thoughts discriminating best for moderate to severe depression.

As such, our findings are in line with recent findings concluding that somatic symptoms should be included in the assessment of depressive symptoms among chronic ill patients.^1^, ^2^, ^3^, ^4^, ^5^ Our findings also support previous research^9^ showing the unidimensionality of the PHQ‐9 and therefore warrant using a total score. Previous studies investigating distinct depressive symptoms have mostly dichotomized depression,^1^, ^5^ and so our study adds to previous research by showing that somatic symptoms sleep problems and fatigue mainly discriminate for mild depression, whereas somatic symptoms appetite changes and concentration difficulty discriminate evenly across depression severity.

### 4.1 Clinical implications

Our findings have important clinical implications, as results suggest that the screening purpose determines the utility of items. If a clinician or researcher is interested in distinguishing moderate to severely (PHQ≥10) from not to mildly depressed cancer patients (PHQ<10), the PHQ‐2 questionnaire may be a valuable screening tool, consisting of the two (affective) core symptoms “anhedonia” and “depressed mood,” as these discriminate properly for moderate depression. However, if a clinician or researcher wants more insight into the overall depression levels in cancer patients, then the PHQ‐9 questionnaire seems more appropriate, since it has high discriminative properties in terms of differentiating mild, moderate, and severe depression.

### 4.2 Study limitations

A major study limitation involves the absence of a golden standard (ie, diagnostic interview). Yet, it should be noted that even when applying such an interview, distinguishing whether somatic symptoms originate from depression or from cancer (treatment) remains challenging for patients and professionals, so a possible overlap in some degree is inevitable when assessing depressive symptoms among chronic ill patients. Future research is needed to repeat our analyses in healthy persons to examine whether our finding of high discriminating value of somatic symptoms for mild depression is (not) caused by an underlying somatic illness.

In conclusion, our study results point out that both somatic and cognitive‐affective symptoms of the PHQ‐9 are valuable in measuring symptoms of depression in cancer patients and that the screening purpose determines the relative utility of distinct symptoms.

## CONFLICT OF INTEREST

The authors have no potential conflicts of interest to report.

## ETHICS STATEMENT

The study was approved by the Medical Ethical Committee of the University Medical Center Groningen (METc 2014/214).
